# RURALITY, ALCOHOL, AND OPIOID USE AS PRECURSOR TO ELDER ABUSE

**DOI:** 10.1093/geroni/igz038.2802

**Published:** 2019-11-08

**Authors:** Faika Zanjani

**Affiliations:** Virginia Commonwealth University, Richmond, Virginia, United States

## Abstract

Pain, alcohol, and opioid risk management is an increasingly important issue in the care of the older adult population. Older adults are at higher risk for pain and the negative outcomes associated with opioid and alcohol use due to higher prevalence of multimorbidity, polypharmacy, and the age-associated changes in metabolism and elimination. Evidence-based approaches for pain and substance use management exist and are effective for older adults, but underutilized and have limited availability in rural areas. Therein, undermanagement of pain and substance use becomes a risk factor for elder abuse. Improved dissemination and implementation in practice, as well as training for health professionals and the community is needed to better realize and prevent negative health outcomes from pain, alcohol, and opioid use. Community education and training tools for health professionals will be discussed, with suggestions for community initiatives to address pain, alcohol, and opioid risk management to prevent elder abuse.

